# Hardness, an Important Indicator of Bone Quality, and the Role of Collagen in Bone Hardness

**DOI:** 10.3390/jfb11040085

**Published:** 2020-12-01

**Authors:** Ahmed Ibrahim, Nicole Magliulo, James Groben, Ashley Padilla, Firas Akbik, Z. Abdel Hamid

**Affiliations:** 1 Mechanical Engineering Department, Farmingdale State College, Farmingdale, New York, NY 11735, USA; magln@farmingdale.edu (N.M.); jpgroben@gmail.com (J.G.); 2 Biology Department, Farmingdale State College, Farmingdale, New York, NY 11735, USA; padiad@farmingdale.edu; 3 Chemistry Department, Hofstra University, Hempstead, NY 11549, USA; fakbik1@pride.hofstra.edu; 4 Central Metallurgical Research and Development Institute, Helwan 11421, Egypt; forzeinab@yahoo.com

**Keywords:** bone quality, bone strength, Vickers microhardness, bone hardness, bone heterogeneity, collagen fibrils, nanoindentation of bone, organic matrix degradation

## Abstract

Bone is a nanocomposite material where the hard inorganic (hydroxyapatite crystallites) and organic (collagen fibrils) components are hierarchically arranged in the nanometer scale. Bone quality is dependent on the spatial distributions in the shape, size and composition of bone constituents (mineral, collagen and water). Bone hardness is an important property of bone, which includes both elastic and plastic deformation. In this study, a microhardness test was performed on a deer bone samples. The deer tibia shaft (diaphysis) was divided into several cross-sections of equal thickness; samples were prepared in untreated, boiled water treatment (100 °C for 30 min) and sodium hypochlorite (NaOCl) treatment conditions. Microhardness tests were performed on various regions of the tibial diaphysis to study the heterogeneous characteristics of bone microhardness and highlight the role of the organic matrix in bone hardness. The results indicated that boiled water treatment has a strong negative correlation with bone hardness. The untreated bone was significantly (+20%) harder than the boiled-water-treated bone. In general, the hardness values near the periosteal surface was significantly (23 to 45%) higher than the ones near the endosteal surface. Samples treated with NaOCl showed a significant reduction in hardness.

## 1. Introduction

Bone is a biological material characterized by a hierarchical structure [1,2,3,4,5]. It is composed of 50 to 70% mineral, 20 to 30% organic matrix (mainly Type I collagen), and 10 to 20% water. The inorganic component is composed mostly of hydroxyapatite and gives bone its strength and stiffness, whereas the organic component provides elasticity to the bone [3,4,5,6]. The unique mechanical properties of bone arise due to its hierarchical structure at the nanoscale, where nanostructured deformation mechanisms of the collagen microfibrils originate. The fibrils are composed of collagen molecules that consist of a triple helix approximately 300 nm in length and 1.5 nm in diameter [4]. The bones’ inorganic and organic matrices are highly structured into two different tissue types: cortical bone (compact bone) and trabecular bone (cancellous or spongy bone). These two types are classified based on porosity and unit microstructure. Cortical bone is much denser than cancellous bone and has a porosity ranging from 5 to 10%. Cortical bone mainly exists in the shafts of long bones and forms the outer covering around cancellous bone at the ends of joints as well as in the vertebrae. Trabecular bone is highly porous with porosity ranging anywhere from 50 to 90%. 

Bone strength is a result of a complex interactions of material properties (organic and inorganic) and structural properties (geometry and distribution) [4,5,6]. Bone fractures and fragility are directly related to lower bone strength. The relative amounts of the inorganic mineral and the organic matrix are the main foundation of the microstructure of bone at both the microscopic and macroscopic levels and profoundly determine its mechanical strength [6,7,8,9,10]. The mechanical properties of bone reflect the intrinsic material properties of its constituents and the way in which they are arranged and interact. 

Bone is inherently heterogeneous and anisotropic owing to spatial distribution variations in the shape, size and composition of its constituent building blocks [3,4,5,6]. As a result of the hierarchical nature of bone, heterogeneity is expected to exist at multiple length scales [6]. Macroscopically, significant variations in mechanical properties have been observed for different anatomical locations, as well as for regions within a particular anatomical location [9,10,11,12,13]. Hardness indentation studies at the microscopic level have identified large variations in moduli and hardness for specific features such as trabeculae and lamellae in osteons. These variations have been attributed to collagen fibril orientation and variations in mineral content induced by remodeling [14,15,16,17,18]. Heterogeneity can have a positive effect on bone toughness, consequently increasing bone fracture resistance [6]. Collagen cross-link formation is thought to affect the mechanical properties of bone at a material level [19,20]. The heterogeneity of bone porosity, collagen fiber orientation, density and mineralization lead to a gradient of bone material properties and can have a strong effect on the structural performance of bone [21,22,23,24,25,26,27,28,29]. 

The degree of mineralization of bone (DMB) matrix is a key determinant of bone strength at the tissue level [7,8,9]. The mineral content of bone directly correlates with Young’s modulus and stiffness in both cortical and trabecular bones [7,10,12,18,30,31,32]. Previous studies showed significant differences in the indentation modulus and microhardness between different osteons [11]. Newly formed osteons had a lower modulus (34%) and hardness (41%) than older osteons in femoral cross-sections. Statistically significant differences in DMB were reported between different bone types (trabecular vs. cortical bone) [33,34,35,36,37] and between different cortical regions of the same bone.

Degree and distribution of mineralization and composition of trabecular and cortical bone have a direct effect on the hardness and mechanical properties of bone [38,39]. Collagen is an integral part of bone structure, giving the properties of toughness, strength and elasticity. The effects of collagen on bone strength and hardness have been demonstrated in many studies [4,8,19,40,41]. 

**Figure 1:**
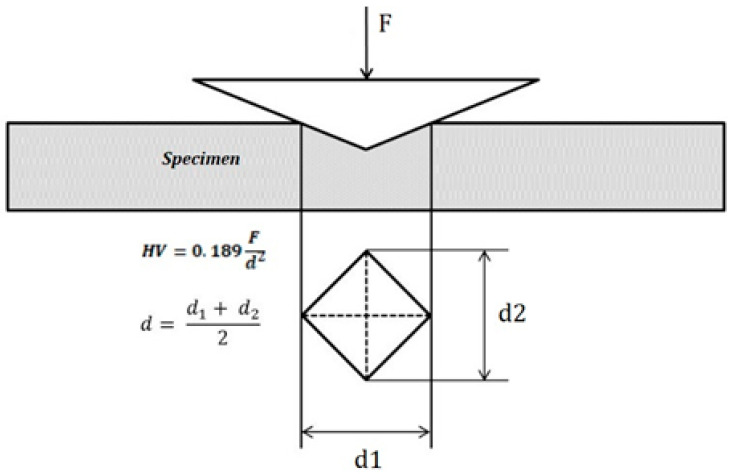
Vickers microhardness test.

Bone hardness is one of the most important properties of bone, which encompasses elastic deformation and plastic deformation. Hardness is a measure of a material’s resistance to deformation by indentation. Hardness has long been a property of considerable importance in engineering materials. Both hardness and strength are important properties of materials and they often obey the three-times empirical relationship: Hv = 3 σ_y_, where σ_y_ is the yield strength for metals and Hv is a Vickers number. However, the relationship between strength and hardness for biological materials does not follow the above formula exactly. The basic principle of the microhardness test is the application of a force via a diamond indenter that results in an indentation on the surface of the specimen. Hv is determined from the force and the area of the indentation (Figure 1). Vickers microhardness (Hv) is a common test that uses a Vickers diamond indenter to measure the hardness of materials.

Microhardness and nanoindentation tests are commonly used tests to assess the hardness of biological materials at the microscale and nanoscale levels [25,26,27,28,29,30,31,32,33,34,35]. Nanoindentation measures the hardness as well as the elastic modulus at the nanoscale level (lamella). Bone hardness was also found to strongly correlate with mineralization [14,33]. Anterior and posterior variations in the mechanical properties of human vertebrae were measured by nanoindentation [35]. There was a statistically significant difference in hardness between the anterior and the posterior regions. It was suggested that the difference is due to a larger amount of mineralization heterogeneity. Indentation techniques have been commonly used in evaluating the fracture toughness and strength of biomaterials and hard tissues [41,42,43,44,45,46,47].

In this study, we investigated the heterogeneous characteristics of bone microhardness in various regions of the tibial diaphysis under different conditions. Microhardness tests were performed on various regions of the tibial diaphysis to study the distribution characteristics of bone microhardness and highlight the role of the organic and inorganic matrices in bone hardness. The effects of thermal degradation and solution bleaching on the organic matrix and the hardness of the bone are also investigated.

## 2. Materials and Methods

### Bone Sample Preparation

Tibia bones of white-tailed deer were obtained from a local processing factory. All soft tissues were removed and the bones were stored at −20 °C. Bone samples were divided into four groups: G I (untreated bone); G II (treated with boiled water at 100 °C for 30 min, G III (soaked in NaOCl (Clorox bleach −7.5%) for 1 and 2 h) and G IV (untreated cancellous bone). Before testing, bones were soaked in a 3% hydrogen peroxide (H_2_O_2_) solution for 24 h. The cross-section of the samples was prepared in accordance with ASTM E384. First, the main shaft (diaphysis) of the bone was sectioned into 0.250-inch-thick segments using a Buehler-ISOMET 4000 (Buehler Inc., Lake Bluff, IL, USA) precision saw equipped with a diamond blade. The cleaned cross-section was then cold-mounted using epoxy resin. After mounting, the samples were subjected to automatic grinding using a Buehler-AutoMet Grinder-Polisher (Buehler Inc., Lake Bluff, IL, USA) in steps (180, 240, 800 and 1200 grit). The bone samples were then polished using a 6 µm and 1 µm diamond suspension liquid to give the samples a mirror-like surface finish. 

Bone microhardness was measured using a Mitutoyo Digital Vickers Microhardness Tester (HM123) (Mitutoyo Corp, Aurora, IL, USA) with a 50 g load and a 10 s dwell time. Measurements were taken in Vickers hardness number (VHN) in accordance with ASTM E384 [18]. Every measurement recorded was an average of three measurements. A minimum distance of 3d (diagonal of indentation) was kept between any two consecutives indentations. The tested sample was highly polished and flat. The maximum deviation in flatness was less than 0.004 mm. 

## 3. Results & Discussion

### 3.1. Vickers Microhardness Testing

**Figure 2:**
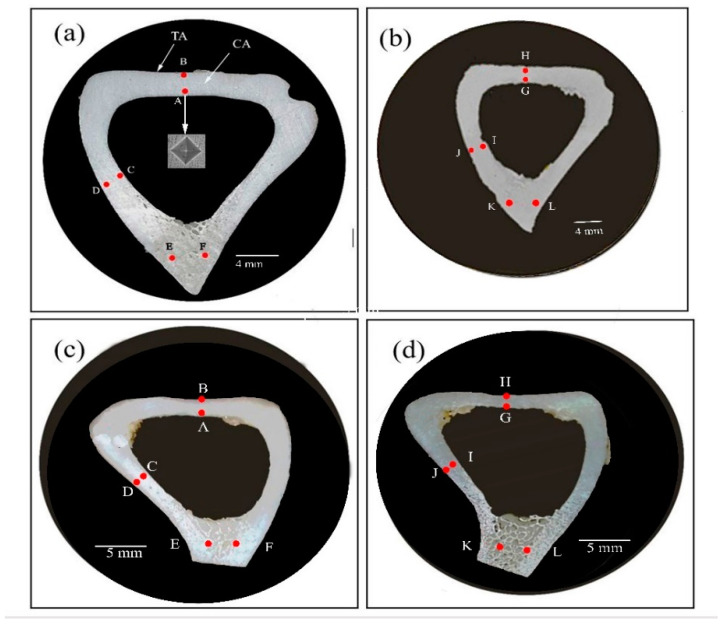
Cross-sectional views of the diaphysis of a deer tibia bone. G I (**a**,**b**): untreated; G II (**c**,**d**): boiled at 100 °C for 30 min. A–L are selected indentation locations in this study.

The microhardness measurements of the untreated bone (sample G I) are shown in Table 1 for the locations (a and b) shown in Figure 2. The mean hardness values ranged from 64.4 Hv on Sample a (from A to F) to 60.1 Hv on Sample b (from G to L). However, there were significant variations between the hardness values near the endosteal (inner) surface (A, C, G, I) and near the periosteal (outer) surface (B, D, H, J). The hardness value at Location B was ~23% higher than at Location A and the hardness value at Location D was ~45% higher than at Location C. These variations are statistically significant and indicate a fundamental change in the bone microstructure at these locations. 

The importance of these results lies in the fact that these significant changes in hardness values took place within a very short distance (1.5 to 2 mm). This is likely due to variations in the mineral content of the extracellular matrix resulting from bone remodeling activities at the endosteal and periosteal surfaces. This result indicated that deer bone exhibits heterogeneity in hardness that varies depending on the location. Heterogeneity refers to the spatial variation in the structure and properties of materials. Bone is a hierarchical structure and exhibits heterogeneity at multiple length scales [3,4]. It is worth noting that an increase in bone hardness at the periosteal surface tends to increase bone bending strength.

The microhardness measurements of the boiled-water-treated bone (sample G II) are shown in Table 1 for the locations (c and d) in Figure 2. The mean hardness value ranged from 53.6 Hv for Sample c (from A to D) to 58.25 Hv for Sample d (from E to H). The hardness values at Locations A to D and E to H for Samples c and d revealed a similar trend to the results seen in Samples a and b above. For example, the hardness value at Location A was ~20% higher than at Location B. Again, the hardness values measured on the periosteal surface of the bone cross-section were significantly higher than the measurements on the endosteal surface. The main point here is that hot water induces some thermal degradation of collagen and had a negative effect on the hardness of the bone. The untreated bone, Sample a, was ~20 % harder than the hot-water-treated Sample c. 

**Figure 3:**
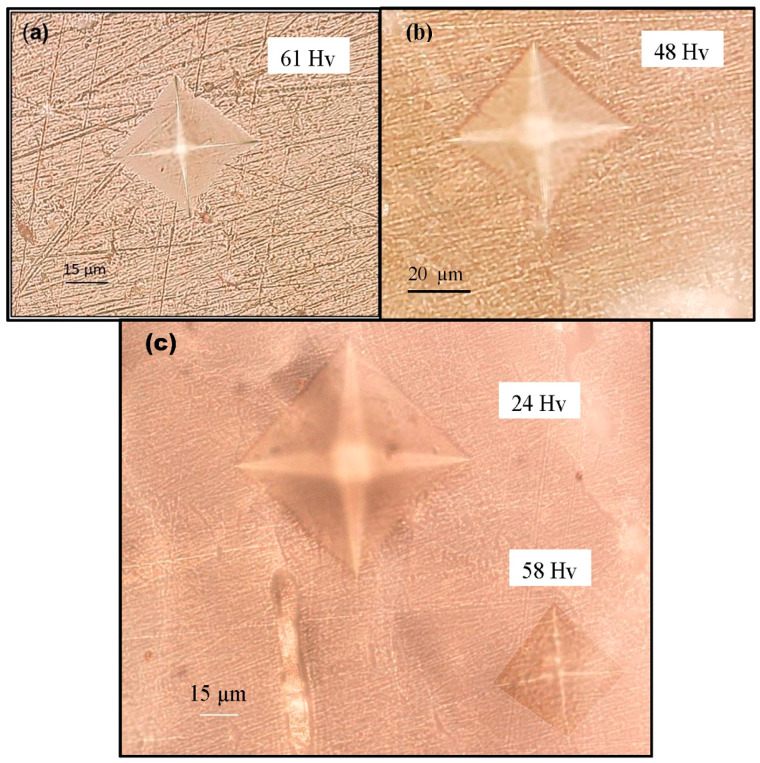
Bone surface indentations obtained by Vickers hardness test. (**a**) Untreated sample; (**b**) sample treated with sodium hypochlorite for 1 h; (**c**) sample treated with sodium hypochlorite for 2 h.

Water occupies ~10 to 20% of the volume in bone and is located both within pores and bound to the matrix [48,49,50,51,52]. The water bound to the matrix stabilizes the collagen structure, binding molecules through hydrogen bonds [51]. The mechanical properties of bone have long been known to depend on the degree of hydration, and bound water contributes to bone toughness [53,54]. The interactions of water with the collagen play a significant role in bone toughness. Boiled water weakens this bond, consequently softening collagen’s strength and hardness. Previous studies indicated that thermal degradation of the organic matrix resulted in decreased elasticity and toughness [55,56,57,58,59,60]. It was suggested that thermal denaturation of collagen involving the rupture of hydrogen bonds usually start at 65 ± 10 °C. As we mentioned before, the effect of collagen on bone strength has been demonstrated in many studies [15,19,40]. Thus, our result is in line with many experimental studies that highlighted the role of collagen in strengthening the bone. In fact, in our recent research (not published yet), we have shown (Figure 3) that boiled-water-treated turkey femur had a detrimental effect on bone toughness. It is interesting to note that the elastic modulus did not change indicating that the DMB also did not change. Fantner et al. [61] investigated the changes in bone’s properties due to heat-induced degradation of the organic matrix. They show that heat treatment changes the microfracture of trabecular bone. They indicated that the failure mode of untreated trabecular bone was fibril-guided delamination; the boiled bone fractured with many small filaments spanning the microcracks indicating that the collagen had softened and lost some elasticity. The baked (200 °C) trabecular bone fractured with no filaments spanning the crack, a clear indication of total degradation of the organic matrix. This result confirms that thermal denaturation of collagen has a strong negative effect on the fracture strength of collagen. 

### 3.2. Effect of Chemical Treatment on Bone Hardness

NaOCl is a frequently used irrigant as it is an excellent organic tissue solvent [62,63]. Several studies have investigated the degradation of bone organic matrix in a NaOCl solution [64,65,66]. NaOCl molecules can oxidize the organic matrix, denature the collagen and adversely affect the hardness of bone

Figure 3b shows bone samples treated with NaOCl for 1 h. The Vickers hardness of the treated sample was 48 Hv (21% reduction) compared with the untreated sample (Figure 3a) at 61 Hv. Moreover, the effect of NaOCl on the morphology of the treated sample is clearly seen in Figure 3b. Figure 3c shows Vickers indentations of bone treated for 2 h. The time-dependent effect of NaOCl on bone morphology and hardness is clearly visible. The Vickers hardness of the treated sample was 24 Hv compared with the original Vickers indentation of 58 Hv before the treatment. This is a significant (58%) reduction in bone hardness. The large amount of indentation in treated bone clearly indicates that bone is much softer as a result of the significant loss of minerals. 

The results above demonstrate significant degradation of the organic and inorganic matrix as a result of NaOCl solution treatment. The effect of NaOCl in the first hour was mainly on the collagen present on the bone surface. In the second hour of treatment, NaOCl started to penetrate deeper and the minerals started to deteriorate quickly, which was clearly seen in the significant reduction in the hardness. 

**Figure 4:**
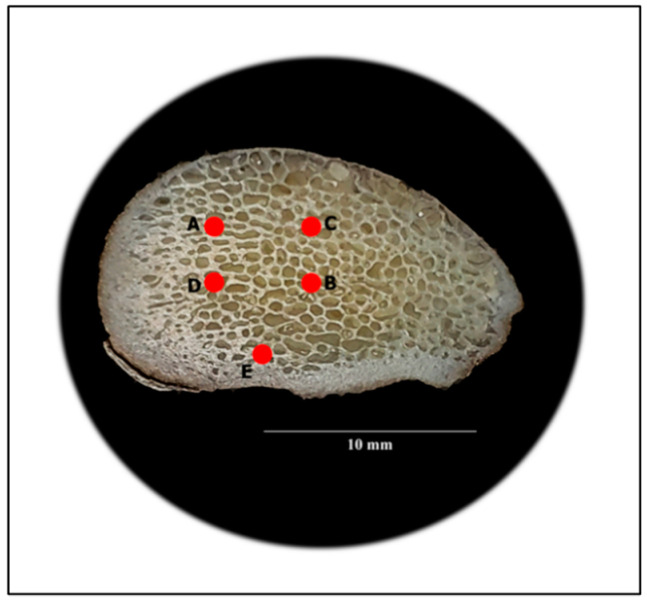
Cross-section of the medial condyle of a femur (sample G IV), showing the cancellous bone. A–E are selected indentation locations in this study.

Figure 4 shows sample G IV (untreated cancellous bone) from the medial condyle. The bone hardness measurements are shown in Table 2. The mean hardness value was 65.0 Hv, which is very compatible with the 64.4 Hv measured on the untreated cortical sample, G I. It is interesting to note that the hardness of the cancellous bone, which has a very high porosity (50 to 90%), is of the same magnitude as the hardness of the cortical bone (sample G I), which has very low porosity (5 to 10%). More importantly, the cancellous bone typically has ~10% less calcium [36]. Several studies have shown that human cortical bone hardness is 10–20% higher than that of trabecular bone [28,36]. The cancellous bone of the femoral head is normally exposed to high levels of compressive and tensile stresses. This is certainly true in animals such as deer. Deer can sprint as fast as 35 miles per hour and are also great jumpers. Our hardness results clearly indicate that deer are well adapted to resist the high bending stresses at the joints. Finally, hardness can be considered an indicator of the mechanical competence of cancellous bone.

## 4. Conclusions

In this study, we have demonstrated that the hardness of deer bone is heterogeneous across the bone cross-section, most likely due to variations in the degree of mineralization and collagen. In addition, we have demonstrated that the organic matrix (collagen) is an important determinant of bone hardness. We have showed that thermal degradation of collagen by heat or a solvent has a strong negative effect on the microhardness of bone tissue.

## Figures and Tables

**Table 1:** Vickers microhardness of groups G I (a and b) and G II (c and d).

Location	Microhardness (Hv) G I (a & b)	Location	Microhardness (Hv) G II (c & d)
A	56.7 ± 4.5	A	48.0 ± 4.7
B	69.8 ± 3.2	B	58.1 ± 3.9
C	53.3 ± 4.8	C	49.8 ± 3.3
D	77.7± 6.3	D	58.6 ± 4.4
E	65.8 ± 5.6	E	48.6 ± 4.6
F	62.9 ± 4.1	F	53.0 ± 5.3
G	55.0 ± 3.0	G	56.2 ± 3
H	66.3 ± 5.3	H	62.5 ± 5.0
I	53.7 ± 4.2	I	57.3 ± 2.6
J	67.2 ± 6.7	J	63.7 ± 4.5
K	62.2 ± 5.6	K	46.4 ± 3.8
L	56.1 ± 4.3	L	48.3 ± 4.1

**Table 2:** Vickers microhardness values (Hv) of the cancellous bone.

Location	Microhardness (Hv)
A	66.8 ± 5.6
B	52.2 ± 5.8
C	76 ± 6.3
D	65.2 ± 4.2
E	65 ± 5.5
